# Notes of the Week

**Published:** 1921-05-28

**Authors:** 


### NOTES OF THE WEEK.

### THE PRINCE AT BRISTOL.

When the Prince of Wales visits Bristol on June 10 he has promised to lay the foundation-stone of the new building of the Bristol Homœopathic Hospital in the grounds of Cotham House. In July 1920 the hospital moved from its old home in Brunswick Square to Cotham House, which was adapted for use as a temporary hospital through the generosity of Mr. W. Melville Wills, the president. The building of the new hospital in the grounds has been begun, and Mr. Wills proposes to carry out the scheme completely. This generosity renders an enormous service to the hospital, and through it to Bristol also. It is interesting to recall that the hospital's quarters in Brunswick Square have been purchased by the Bristol Maternity Hospital Committee.

### A LONG RUN OF LEGACIES.

Sir Robert Finlay, when lately addressing the supporters of the Royal Edinburgh Hospital for Sick Children, made a financial confession and ventured on a medical prophecy. He said that, from the advances made in orthopædics, he thought that the sight of a club foot would be as rare in thirty years' time as a face marked by small-pox at present. In regard to finance, he said that even before the war the hospital had paid one-third of its annual expenses from legacies, and was now paying even more. For the past twenty years the average sum received from legacies was about £7,000, but in the past two years the hospital had received over £31,000. Who could say whether this source of income would continue? Certainly it has been a long run of legacies, and perhaps children's hospitals still tend to attract testators more than other institutions. We hope that the experience of the Edinburgh Royal is not unique and will be maintained.

### DEATH OF MR. WALTER ALVEY.

We deeply regret to record the death of Mr. Walter K. Alvey, which took place on May 21 at his residence at Streatham after an illness which had lasted a year. Mr. Alvey did not give up work till last January, and his death, though not unexpected, has caused sincere grief in the hospital world, where he was well known and deservedly respected. The loss to Charing Cross Hospital, to whose service he devoted upwards of twenty-five years of his life, with marked ability, energy, and enterprise, is very great. First as clerk, then as assistant secretary and accountant, as acting secretary for six months on Mr. Arthur Reade's retirement, and as secretary since January 1907, he had acquired a wide knowledge of the detail of the administrative work of the hospital; his grasp of accounts and liking for figures were of immense assistance in the financial strain and difficulties which Charing Cross Hospital has undergone in recent years, which were so successfully overcome that, in the words of the chairman a few months ago, the whole hospital is open, full from attic to basement, beautifully equipped and free from all money worry. Outside his own hospital Mr. Alvey took a deep interest in all that concerned hospital workers; he was a vice-president of the Incorporated Association of Hospital Officers, and an active worker in its interests, and his eloquent and helpful contributions to its debates will be greatly missed. The sympathy of a large number of people, including many hospital workers, goes out to Mrs. Alvey and the family in their loss.

### CROWDED UNIVERSITIES.

At the institutions themselves it may produce temporary difficulties coincident with overcrowding, but in many ways it augurs well for the future that so great an increase has occurred in the numbers of people undergoing a University training. The present increase may in part be due to the presence of ex-Service students, but it is satisfactory to note that at those centres in receipt of grants from the University Grants Committee there were in 1913-14 22,234 students, whereas the number for 1919-20 is 36,424! And these figures do not include the increases known to exist at Oxford, Cambridge, Trinity College, Dublin, and Guy's Hospital Medical School, which are not in receipt of grants.

### THE GENERAL MEDICAL COUNCIL.

At the meeting of this body on May 24 some interesting points in connection with medical practice were brought forward. Registration of medical students has fallen from 3,420 in 1919 to 2,531 in 1920, but this number is, of course, still higher than in any pre-war year. Sir Donald MacAlister is undoubtedly correct in his opinion that if the number were still further reduced it would be directly in the interest of really sound professional education. He advocates raising the educational standard for admissions and increasing the fees for professional tuition. It is noteworthy that dental registration still falls short of the annual number before the war, but the Bill now before Parliament will probably have a marked effect in this quarter.

### EGG POISONING.

We would point out to the evening paper which finds something remarkable in the case of a man who is unable, without nausea, to eat eggs that such instances are by no means uncommon. Their correspondent on this subject complains that the condition "is a disease, also like hay fever, in which the medical profession takes very little interest." But one must make strong protest against this statement in view of the great amount of investigational work recently performed in connection with the effects of foreign proteins upon the human body. The albumens, etc., of eggs fall naturally into this class, also a variety of sera used for inoculation purposes and some animal proteins concerned in "animal asthma" which is closely allied to so-called hay fever. We would suggest that E. W. M. and others responsible for these comments on "the terrible egg" should glance through any annual record of British medical work and note that the profession does indeed take interest in the phenomenon known as anaphylaxis.

### THOMAS GUY.

Lot No. 352 at a sale of autographs, etc., to be held at Messrs. Puttick and Simpson's rooms in Leicester Square (the house was once the residence of Sir Joshua Reynolds), contains an interesting hospital item in an autograph document signed by Thomas Guy, founder of Guy's Hospital. Included also in the same lot are a portrait and two prints of the great bookseller and philanthropist. The document, it may be added, dates from 1722, the very year that Guy's was founded. In other lots at the same sale autographs and letters are also to be seen of Edward Jenner, the institutor of vaccination, John Radcliffe, Abernethy, Richard Mead, Sir Henry Halford, and other medical worthies.

### DANGEROUS DRUGS.

New Regulations concerning Dangerous Drugs were gazetted on May 24. In their present form the new proposals place medical men, veterinary surgeons, and registered dentists on an entirely separate footing as regards various duties and rules. Dangerous drugs may be lawfully in their possession and they may prescribe or dispense them. Also their day-book records are deemed to be satisfactory records. The rule making necessary a statement on the bottle of the amount of drug present does not now apply to preparations dispensed by doctors or from their prescriptions. Opium, morphia, cocaine, and heroin are included, but not so some well-known official preparations, such as Dover's powder, etc. These Regulations, upon which we hope to comment more fully next week, would come into force on September 1.

### THE CONTROL OF FEVERS.

To those who need further evidence of the value of organised preventive measures against disease, we would quote the statement made by Dr. Wm. Hunter, of the London Fever Hospital, in his recent Chadwick Lecture. At the moment the death-rate from fevers is the lowest in the history of England, and the lowest of any country in the world. He added that with us typhus, typhoid, cholera, smallpox, and scarlet fever are now almost completely under control, the first four being on the point of extinction. Influenza alone shows a true increased prevalence, and that only when we take into account the great epidemic of 1918-19.

### THE VALUE OF MINOR ALLIES.

Few auxiliary societies can show a better result than the Nottingham Sick Children's Hospital Cot and Free Medicine Fund. In 1902, when it began, the object of the fund was to provide £30 a year to maintain a cot at the Children's Hospital. The fund has now given to the hospital £3,470, the proceeds of the past year, one-third of which is "to help to clear the hospital from debt." The total grant is £1,200 more than that made by the Society in the previous year. It shows also that auxiliary bodies are always to be encouraged, for little limit can be placed upon the possibility of their expansion, and a linen league, or a cot fund, as in Nottingham, may become an important source of income. Mr. T. Bosworth, the chairman of the Central Committee, and Mr. F. Watson, the hon. secretary, deserve every congratulation upon the remarkable fruits of their work.

### THE EASTBOURNE HOSPITAL INQUIRY.

The special committee appointed to investigate and report on the management of the Princess Alice Hospital, Eastbourne, has reported to a meeting of the governors. The inquiry arose from the allegations of Mr. J. T. Helby, but the committee report that a staff of forty-eight is not too large for the daily average of sixty-two patients, partly because the wards are scattered, and partly because the staff must be prepared for the full number of patients, which is seventy-six. Mr. Helby, who was one of the special committee, did not sign the report. The chairman of the hospital is Mr. Sydney Hudson, who has held the post for twelve years, and has given generously to the institution. The familiar suggestion that the hospital has become "a one-man show" has been made, but the inquiry has now been held, and the Mayor, Mr. E. Duke, who presided at the meeting, declared that the matter had been fully ventilated.

### A CRITICISM OF CO-OPERATIVE BUYING.

In the course of a speech describing the proceedings of the recent meeting of the Midlands Regional Committee of the British Hospitals' Association, Mr. Holloway told the supporters of the Herefordshire General Hospital that experience is against the co-operative purchase of drugs and dressings. It is stated that wholesale merchants are opposing these proceedings, and intend to do so by raising their prices. It seems on the face of it unlikely, in view of the present state of trade.

### INSTITUTIONAL POACHING.

In the latest quarterly paper issued by the Liverpool Council of Voluntary Aid, mention is made of appeals in the city for institutions at a distance, and especially of one for a London hospital during Grand National Week. It is stated that all the local work required was finally undertaken by a Liverpool charity in return for half the proceeds. Criticism of the plan is implied rather than expressed, and probably the only defence would be that the Grand National is almost, as its name implies, a national meeting. Still, the policy is open to question, and it is doubtful if the proceeds received justify the misunderstandings likely to be occasioned by it. St. Dunstan's is mentioned as a competing charity, and the Council has been asked to consider the matter at a time when Liverpool and its charities are feeling the industrial depression. A scheme is suggested on lines made familiar by King Edward's Hospital Fund for London and by the practice of certain American towns for a fund for the general benefit of Liverpool charities. In America the plan is said to have added enormously to the number of subscribers; and it is, of course, the number, rather than the generosity, of those who do subscribe which is lacking.

